# Modeling within-host and aerosol dynamics of SARS-CoV-2: The relationship with infectiousness

**DOI:** 10.1371/journal.pcbi.1009997

**Published:** 2022-08-01

**Authors:** Nora Heitzman-Breen, Stanca M. Ciupe

**Affiliations:** Department of Mathematics, Virginia Polytechnic Institute and State University, Blacksburg, Virginia, United States of America; Columbia University Medical Center: Columbia University Irving Medical Center, UNITED STATES

## Abstract

The relationship between transmission of severe acute respiratory syndrome coronavirus 2 (SARS-CoV-2) and the amount of virus present in the proximity of a susceptible host is not understood. Here, we developed a within-host and aerosol mathematical model and used it to determine the relationship between viral kinetics in the upper respiratory track, viral kinetics in the aerosols, and new transmissions in golden hamsters challenged with SARS-CoV-2. We determined that infectious virus shedding early in infection correlates with transmission events, shedding of infectious virus diminishes late in the infection, and high viral RNA levels late in the infection are a poor indicator of transmission. We further showed that viral infectiousness increases in a density dependent manner with viral RNA and that their relative ratio is time-dependent. Such information is useful for designing interventions.

## Author summary

Quantifying the relationship between SARS-CoV-2 dynamics in upper respiratory tract and in aerosols is key to understanding SARS-CoV-2 transmission and evaluating intervention strategies. Of particular interest is the link between the viral RNA measured by PCR and a subject’s infectiousness. Here, we developed a mechanistic model of viral transmission in golden hamsters and used data in upper respiratory tract and aerosols to evaluate key within-host and environment based viral parameters. The significance of our research is in identifying the timing and duration of viral shedding, how long it stays infectious, and the link between infectious virus and total viral RNA. Such knowledge enhances our understanding of the SARS-CoV-2 transmission window.

## Introduction

The transmission of the severe acute respiratory syndrome coronavirus 2 (SARS-CoV-2), the agent that causes coronavirus disease 2019 (COVID-19), is dependent on the amount of infectious particles present in the environment surrounding the susceptible host, and/or on the proximity between the susceptible and infectious host. Experimental studies that use real-time reverse transcription-polymerase chain reaction (PCR) assays have reported the presence of SARS-CoV-2 RNA in contaminated environmental surfaces [1–4] and in aerosols [5–8]. Moreover, *in vivo* cell culture assays, have shown that particles released into the environment are replication-competent [9] and they can stay infectious in the aerosols for up to three hours [10].

Once an infection is established, the dynamics of SARS-CoV-2 in the upper respiratory tract (URT) is dictated by the interplay between the virus fitness and host immune responses. The total RNA to infectious virus ratio changes over the course of the infection, varying between 10^3^ : 1 and 10^6^ : 1 RNA to plaque forming units (PFU) [11, 12]. Determining the within-host mechanistic interactions responsible for the temporal changes in infectious to non-infectious viral dynamics is important for guiding interventions.

Over the last two years, within-host mathematical models developed for influenza and other respiratory infections have been modified for SARS-CoV-2 infections [13–18]. These models divided total viral titers into infectious and non-infectious particles [19, 20], fitted their sum to total RNA values measured by PCR (used as a proxy for total virus load) and used the results to determine the mechanisms of viral expansion and loss. The models were subsequently used to provide insights into the relative fitness of variants, types of drug interventions [13–15], the relationship between individual infection and population transmission, and the effect of this relationship on testing and vaccine strategies [21, 22]. In most studies, the total RNA to infectious virus ratio in URT is assumed constant over time. However, Goyal *et al*. [23] and Ke *et al*. [13] proposed a nonlinear correspondence between infectious virus and total RNA, and found that a density dependent function best describes their relationship [13].

While there is a reasonable understanding of the mechanistic dynamics modulating SARS CoV-2 infection in the URT, the temporal shedding into the environment of viral RNA and infectious virus has not been explored. Here, we expand a within-host mathematical model to include the dynamics of viral RNA and infectious virus titers in both URT and aerosols. We validate the models against two URT and one aerosol inoculation study in golden hamsters [24, 25] and use the models to determine the relationship between infectious virus and total RNA in both environments. Lastly, we investigate the link between the infectious virus shed in the environment and the probability of a nearby host getting infected. The results can guide interventions.

## Materials and methods

### Experimental data

We use previously published temporal SARS-CoV-2 RNA and infectious virus titer data from two inoculation studies in golden Syrian hamsters:

- *Sia et al. study* [25]: six donor hamsters (all male) were inoculated intranasally with 8 × 10^4^ tissue culture ineffective dose (TCID_50_) of SARS-CoV-2. At 24 hours after inoculation, each donor was transferred to a new cage and co-housed with one naive male hamster. Viral RNA and infectious virus titers were collected every other day (in RNA/ml and TCID_50_/ml) for the first 14 days in both donors and contacts and their weight changes were monitored daily. We will refer to these two groups as donors and contacts.
- *Hawks et al. study* [24]: eight hamsters (4 males and 4 females) were inoculated intranasally with 10^5^ PFU (1.4 × 10^5^ TCID_50_) of SARS-CoV-2. Viral RNA and infectious virus titers were collected daily from nasal washes (in RNA/wash and PFU/wash) and from the exhaled breath (aerosols) (in RNA/hour and PFU/hour) for the first five days and then again at day 10. Since the nasal wash and air samples were collected in 100 *μl* and 400 *μl*, we rescaled the RNA/wash data to RNA/ml by multiplying it with 10 and 2.5, respectively. Moreover, since 1 TCID_50_ = 0.7 PFU, we rescaled the PFU/wash infectious virus titers in the nasal washes and in the air to TCID_50_/ml by multiplying them with 10/0.7 and 2.5/0.7, respectively. Hamsters were weighed daily and the study was terminated when clinical signs of illness were observed. We will refer to these two groups as males and females.

### Within-host and aerosol model

**Fig 1:**
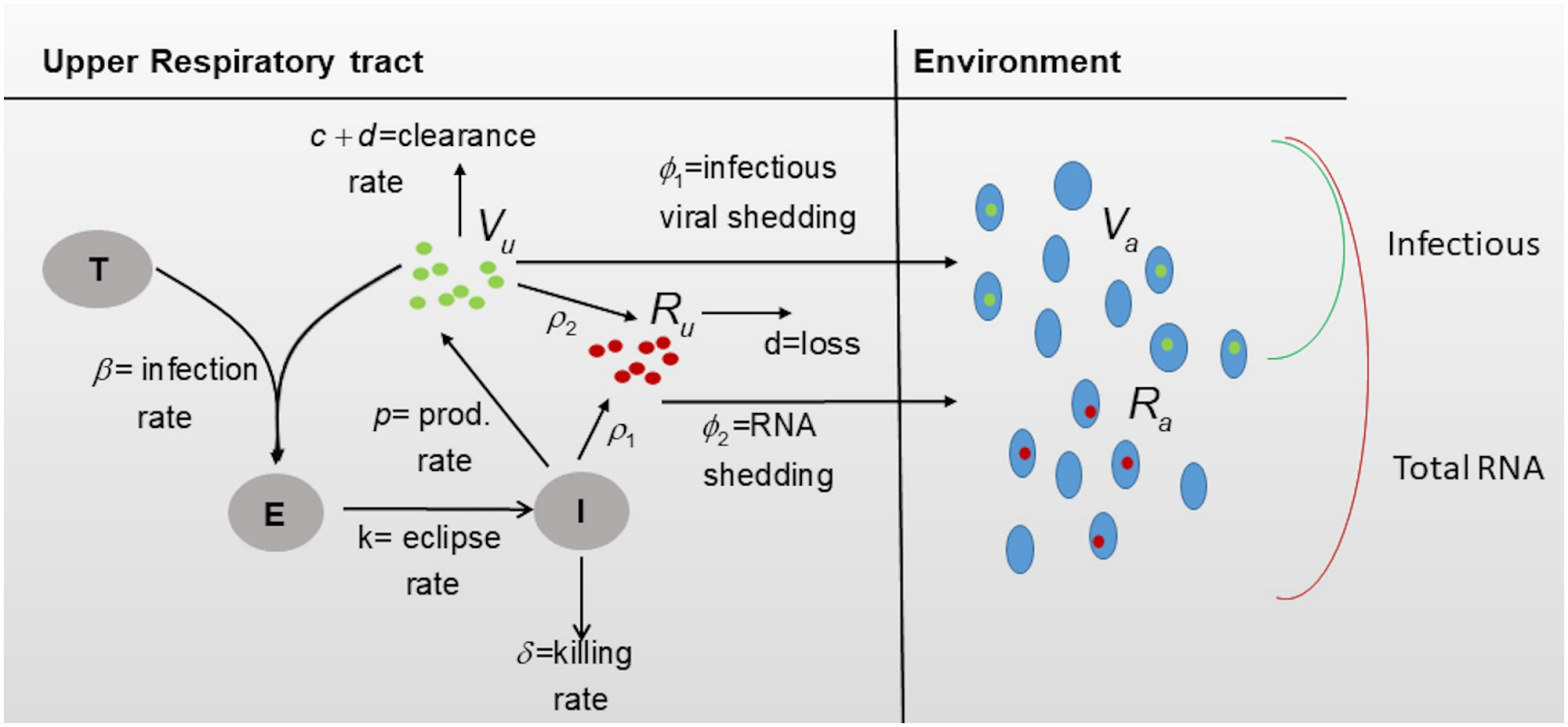
Model diagram. Diagram for model Eqs (3) and (4).

We model the interaction between target epithelial cells *T*, exposed epithelial cells *E*, infected epithelial cells *I*, infectious virus in upper respiratory tract *V*_*u*_, infectious virus in the air *V*_*a*_, total viral RNA in upper respiratory tract *R*_*u*_, and total viral RNA in the air *R*_*a*_ (see Fig 1 for a description). We assume that target cells get infected at rate *β* and become productively infected at rate *k*. Productively infected cells produce infectious virus at rate *p* and die at rate *δ*, due to immune mediated responses. Infectious virus particles in the upper respiratory tract are removed at rate *d* + *c*, where *d* is degradation rate and *c* is the immune clearance rate. Infectious virus is shed into the air at rate *ϕ*_1_, where it loses infectiousness at rate *d* + *d*_1_, where *d* is the degradation rate (as before) and *d*_1_ accounts for enhanced inactivation due to the elements. The equations describing these interactions are

$$\begin{array}{c} \begin{array}{cc} \frac{dT}{dt} & =-\beta TV_{u},\\ \frac{dE}{dt} & =\beta TV_{u}-kE,\\ \frac{dI}{dt} & =kE-\delta I,\\ \frac{dV_{u}}{dt} & =pI-\left(d+c\right)V_{u}-\phi_{1}V_{u},\\ \frac{dV_{a}}{dt} & =\phi_{1}V_{u}-\left(d+d_{1}\right)V_{a}, \end{array} \end{array}$$ (1)

with initial conditions *T*(0) = *T*_0_, *E*(0) = 0, *I*(0) = 0, *V*_*u*_(0) = *V*_0_, *V*_*a*_(0) = 0.

Moreover, we model total viral RNA in the upper respiratory tract *R*_*u*_ and in the air *R*_*a*_ as follows. Since the measured viral RNA may not be a good representation of actual virions (infectious or noninfectious) but also of naked viral RNA from virus neutralization or infected cells death, we assume viral RNA is produced at rate *ρ*_1_ per infected cell, and rate *ρ*_2_ per infectious virus. Rate *ρ*_1_ incorporates both newly synthesized genomic RNA that can be used for replication or transcription, *p*, and naked RNA released from dead infected cells, *q*, so that *ρ*_1_ = *p* + *q*. Rate *ρ*_2_ represents neutralized infectious virus. Genomic RNA particles are removed at rate *d* + *c* (where *d* is the degradation rate and *c* is the immune clearance rate, as before), while naked RNA is lost at rate *d* (with the immune system not being involved in its removal). Lastly, URT total viral RNA is released into the environment at rate *ϕ*_2_ where it is lost at rate *d*. The equations describing total viral RNA dynamics over time are given by

$$\begin{array}{c} \begin{array}{cc} \frac{dR_{u}}{dt} & =\rho_{1}I+\rho_{2}V_{u}-cV_{u}-dR_{u}-\phi_{2}R_{u},\\ \frac{dR_{a}}{dt} & =\phi_{2}R_{u}-dR_{a}, \end{array} \end{array}$$ (2)

with initial conditions *R*_*u*_(0) = *R*_*a*_(0) = 0.

### Parameter values

We assume an initial target cell population in the upper respiratory tract *T*(0) = 10^7^ epithelial cells/ml, as in influenza [26], and no exposed and infected cells *E*(0) = *I*(0) = 0 epithelial cells/ml. The initial infectious virus is given by the inoculum titer, *V*_*u*_(0) = 8 × 10^4^ TCID_50_ for donors and *V*_*u*_(0) = 1.4 × 10^5^ TCID_50_ for males and females. For contacts, who are infected rather than inoculated, *V*_*u*_(0) is unknown. We investigate two scenarios, (i) infection starts with a single infectious virus, *V*_*u*_(0) = 1 TCID_50_, and (ii) infection starts with more than one infectious virus *V*_*u*_(0)>1 TCID_50_, with the exact number being estimated through data fitting. No viral RNA is present in URT at the time of inoculation, *V*_*a*_(0) = 0 TCID_50_, and neither infectious virus nor viral RNA are present in the air at the time of inoculation, *R*_*u*_(0) = *R*_*a*_(0) = 0 TCID_50_. We assume that the infectivity rate is *β* = 5 × 10^−6^ mL/(TCID_50_ x day), infectious virus removal rate is *c* + *d* = 10 per day [13], the eclipse rate is *k* = 4 per day [13], the RNA degradation rate is *d* = 1 per day, the RNA release due to neutralization is *ρ*_2_ = *c* = 9 per day, and the enhanced inactivation due to the elements is *d*_1_ = 1. Lastly, since RNA and infectious virus from exhaled breath are only collected in Hawks *et al*. [24], we assume *ϕ*_1_ = *ϕ*_2_ = 0 in models (1) and (2) when applied to donors and contacts and {*ϕ*_1_, *ϕ*_2_} ≠ 0 when applied to males and females.

### Incorporating weight variability into the model

**Fig 2:**
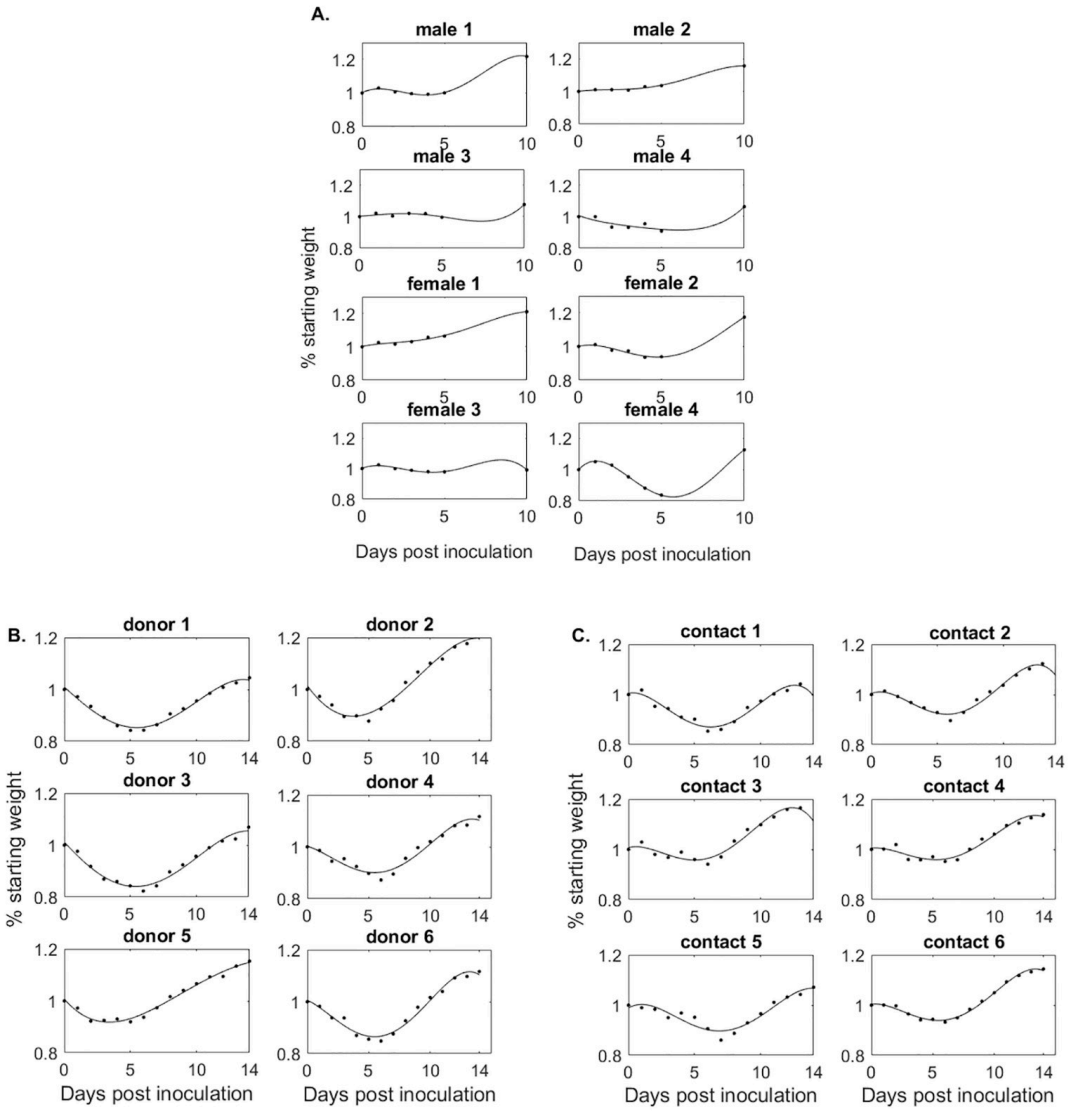
% Weight change from baseline over time. Weight functions (black lines) versus weight data (diamonds) in (A) males and females, (B) donors, and (C) contacts.

The weights of males and females vary from 56 to 76.1 grams (with an average of 70 grams) at inoculation. After challenge, each subject was weighed, as a way to monitor them for clinical signs of illness, and daily percent weight changes were reported as changes from the baseline weight *w*_0_ = 1, 0 ≤ *w*_*i*_ ≤ 1 for days *i* ∈ *S* = {1, 2, 3, 4, 5, 10} post infection. To account for all intermediate time points, we fitted a 4-degree polynomial to the weight data (smallest degree polynomial that gave a residual sum of squares <10^−3^), and the resulting percent weight functions are shown in Fig 2A.

The initial weights of donors and contacts are unknown. After inoculation, each subject was weighed and percent weight changes were reported as changes from the baseline weight *w*_0_ = 1, 0 ≤ *w*_*i*_ ≤ 1 for days *i* ∈ *S* = {2, 4, 6, 8, 10, 12, 14} post infection. As before, we fitted a 4-degree polynomial to the weight data, and the resulting percent weight functions are shown in Fig 2B and 2C for the donors and contacts, respectively.

To include the weight variability into the model, we assume that weight changes result in adjusted available epithelial cells [27]. At day *t* target, exposed and infected cells become *T*_1_(*t*) = *T*(*t*) × *w*(*t*), *E*_1_(*t*) = *E*(*t*) × *w*(*t*), and *I*_1_(*t*) = *I*(*t*) × *w*(*t*). The models for the weight-dependent cell populations become

$$\begin{array}{c} \begin{array}{cc} \frac{dT_{1}}{dt} & =-\beta T_{1}V_{u},\\ \frac{dE_{1}}{dt} & =\beta T_{1}V_{u}-kE_{1},\\ \frac{dI_{1}}{dt} & =kE_{1}-\delta I_{1},\\ \frac{dV_{u}}{dt} & =\frac{p}{w(t)}I_{1}-\left(c+d\right)V_{u}-\phi_{1}V_{u},\\ \frac{dV_{a}}{dt} & =\phi_{1}V_{u}-\left(d+d_{1}\right)V_{a}, \end{array} \end{array}$$ (3)

and

$$\begin{array}{c} \begin{array}{cc} \frac{dR_{u}}{dt} & =\frac{\rho_{1}}{w(t)}I_{1}+\left(\rho_{2}-c\right)V_{u}-dR_{u}-\phi_{2}R_{u},\\ \frac{dR_{a}}{dt} & =\phi_{2}R_{u}-dR_{a}, \end{array} \end{array}$$ (4)

Moreover, the initial conditions become *T*_1_(0) = *ξT*_0_, *E*_1_(0) = 0, *I*_1_(0) = 0, *V*_*u*_(0) = *V*_0_, *V*_*a*_(0) = 0, *R*_*u*_(0) = *R*_*a*_(0) = 0 where *ξ* = {1.06, 1.09, 0.97, 0.81, 1.01, 0.92, 1.094, 1.04} is scaling from the average initial weight of 70 grams for males and females and *ξ* = 1 for donors and contacts [25].

### The basic reproduction number

The basic reproduction number (or basic reproductive ratio) is defined as the number of infected cells (or virus particles) that are produced by one infected cell (or virus particle) when the virus is introduced into a population of uninfected target cells *T*_1_(0) = *ξT*_0_. It is given by

$$\begin{array}{c} R_{0}=\frac{\beta p}{(c+d+\phi_{1})\delta }\xi T_{0}, \end{array}$$ (5)

with *ϕ*_1_ = 0 for donors and contacts.

### Data fitting

Using models (3) and (4) we estimate parameters ${\text{param}}_{w}^{1}=\left\{\delta ,p,\phi_{1},\rho_{1},\phi_{2}\right\}$, by minimizing the functional

$$J\left({\text{param}}_{w}^{1}\right)=J_{V}\left({\text{param}}_{w}^{1}\right)+J_{R}\left({\text{param}}_{w}^{1}\right),$$

where

$$J_{V}\left({\text{param}}_{w}^{1}\right)={\left(\sum_{j\in S}{\left(V_{u}\left(j\right)-V_{u}^{data}\left(j\right)\right)}^{2}+\sum_{j\in S}{\left(V_{a}\left(j\right)-V_{a}^{data}\left(j\right)\right)}^{2}\right)}^{1/2},$$

$$J_{R}\left({\text{param}}_{w}^{1}\right)={\left(\sum_{j\in S}{\left(R_{u}\left(j\right)-R_{u}^{data}\left(j\right)\right)}^{2}+\sum_{j\in S}{\left(R_{a}\left(j\right)-R_{a}^{data}\left(j\right)\right)}^{2}\right)}^{1/2},$$

and *S* = {1, …, 5, 10} days post infection.

Using model (3) we estimate parameters ${\text{param}}_{w}^{2}=\left\{\delta ,p,\rho \right\}$ for donors and either ${\text{param}}_{w}^{2}=\left\{\delta ,p,\rho \right\}$ with *V*_*u*_(0) = 1 TCID_50_ or ${\text{param}}_{w}^{3}=\left\{\delta ,p,\rho_{1},V_{u}\left(0\right)\right\}$ for contacts by minimizing the functional

$$J\left({\text{param}}_{w}^{i}\right)=J_{V}\left({\text{param}}_{w}^{i}\right)+J_{R}\left({\text{param}}_{w}^{i}\right),$$

where

$$J_{V}\left({\text{param}}_{w}^{i}\right)={\left(\sum_{j\in S}{\left(V_{u}\left(j\right)-V_{u}^{data}\left(j\right)\right)}^{2}\right)}^{1/2},$$

$$J_{R}\left({\text{param}}_{w}^{i}\right)={\left(\sum_{j\in S}{\left(R_{u}\left(j\right)-R_{u}^{data}\left(j\right)\right)}^{2}\right)}^{1/2},$$

**Fig 3:**
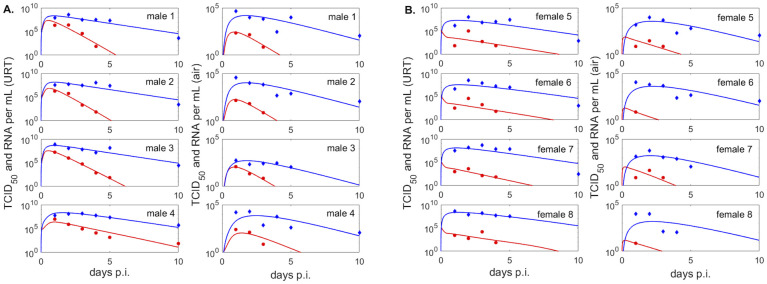
Infectious virus and viral RNA dynamics in upper respiratory tract and exhaled breath of male and female groups. (Left panels) Dynamics of infectious virus *V*_*u*_ (red lines) and viral RNA *R*_*u*_ (blue lines) as given by model Eq 3 versus infectious viral titers (red circles) and RNA (blue diamonds) in the upper respiratory tract of the (A.) males and (B.) females; (Right panels) Dynamics of infectious virus *V*_*a*_ (red lines) and RNA molecules *R*_*a*_ (blue lines) as given by model Eq 4 versus infectious viral titers (red circles) and RNA (blue diamonds) in the exhaled breath of (A.) males and (B.) females. Model parameters are given in Table 1.

**Fig 4:**
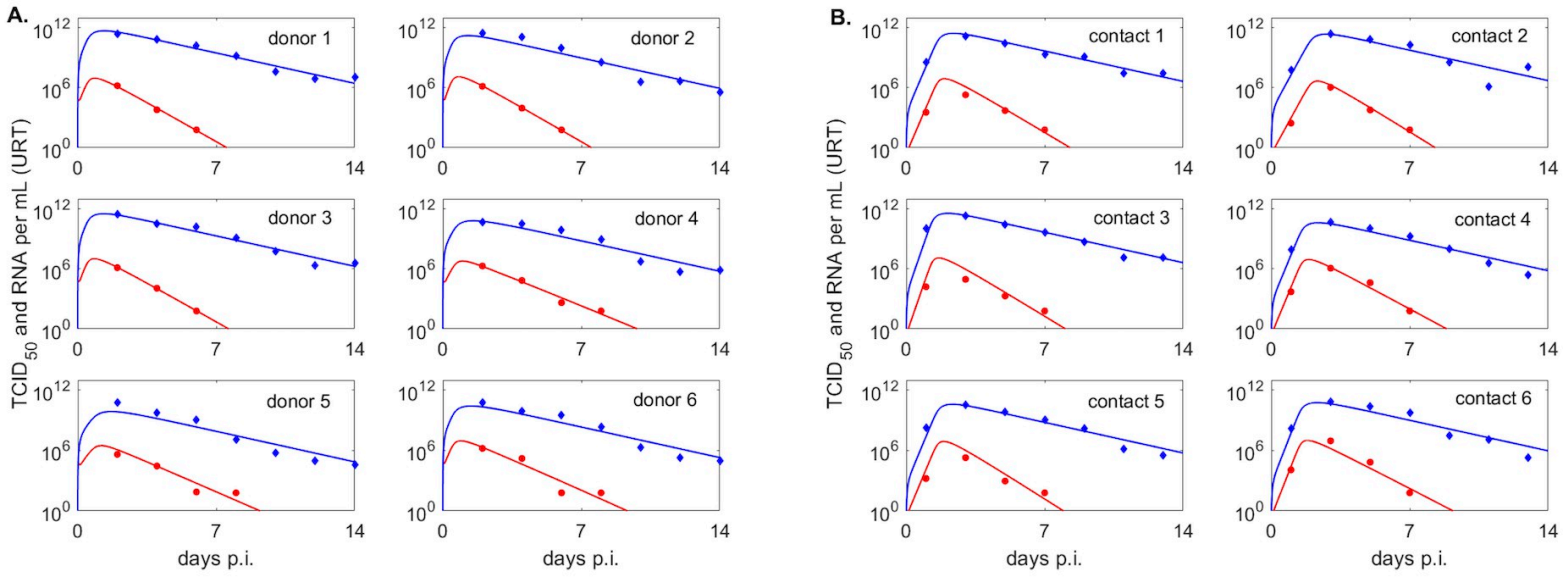
Infectious virus and viral RNA dynamics in upper respiratory tract of donor and contact groups. Dynamics of infectious virus *V*_*u*_ (red lines) and viral RNA *R*_*u*_ (blue lines) as given by model Eq 3 versus infectious viral titers (red circles) and viral RNA (blue diamonds) in the upper respiratory tract of (A.) donors, (B.) contacts with fixed initial virus, *V*_0_ = 1. Model parameters are given in Table 2.

*S* = {2, 4, 6, 8, 10, 12, 14} days post infection for donors, *S* = {1, 3, 5, 7, 9, 11, 13} days post infection for contacts, and *i* = {2, 3}. We only consider the first data point at or below limit of detection. We use the ‘nlinfit’ algorithm in matlab and the resulting estimates for parameter means and 95% confidence intervals are given in Tables 1 and 2. The theoretical solutions of *V*_*u*_ and *R*_*u*_ versus male and female data are shown in Fig 3, versus donor data are shown in Fig 4A, versus contact data when we assume *V*_*u*_(0) = 1 are shown in Fig 4B, and versus contact data when we assume *V*_*u*_(0) is variable are shown in Fig A in S1 Text.

**Table 1:** Individual parameter estimates for male and female groups.

	*p*		*δ*		*ϕ*_1_	× 10^−4^	*ϕ*_2_	× 10^−5^	*ρ*		ssq
male 1	55	(1.1, 2763)	4.3	(2.5, 7.3)	0.5	(0.01, 18)	12	(2, 83)	144	(32, 639)	2.5
male 2	26	(0.5, 1489)	5.2	(2.5, 10.6)	0.7	(0.03, 21)	12	(2, 73)	121	(22, 667)	2.3
male 3	92	(13, 630)	3.5	(2.8, 4.4)	0.1	(0.02, 0.7)	0.2	(0.06, 0.6)	292	(123, 692)	1.4
male 4	8.8	(1.4, 54)	1.5	(1.2, 2.0)	0.7	(0.06, 10)	4.6	(0.6, 33)	88	(20, 374)	2.7
average	45	-	3.6	-	0.5	-	7.3	-	161	-	-
female 5	0.1	-	1.7	(1.0, 3.1)	91	(5, 1600)	26	(2, 450)	81	(7, 945)	3.8
female 6	0.1	-	1.8	(1, 3.1)	20	(0.2,2060)	15	(1, 230)	216	(20, 2291)	3.3
female 7	0.1	-	2.3	(1.3, 3.9)	135	(10,1810)	2	(0.1, 28)	675	(80, 5670)	3.3
female 8	0.1	-	1.6	(1, 3.2)	19	(0.3, 1380)	1	(0.05, 18)	1001	(103, 9716)	2.6
average	0.1	-	1.8	-	66	-	11	-	494	-	-

Individual estimates (mean and 95% confidence intervals) from simultaneously fitting *V*_*u*_, *V*_*a*_ and *R*_*u*_, *R*_*a*_ given by models Eqs (3) and (4) to URT and aerosol infectious virus and RNA data in the males and female groups in Hawks *et al*. [24].

**Table 2:** Individual parameter estimates for donor and contact groups.

	*p*		*δ*		*ρ* × 10^4^		ssq
donor 1	21.7	(0.4–1156)	2.6	(2.0–3.4)	24.8	(6.6–94.1)	1.0
donor 2	29.6	(0.08–10946)	2.6	(1.6–4.0)	8.3	(1.2–56.4)	1.6
donor 3	25.0	(1.3–474)	2.6	(2.0–3.3)	16.5	(4.6–58.9)	1.0
donor 4	13.0	(0.1–1722)	1.8	(1.3–2.7)	2.6	(0.4–18.8)	1.7
donor 5	7.0	(0.01–28711)	1.9	(1.3–2.8)	0.3	(0.01–8.7)	2.2
donor 6	26.3	(1.8–388)	2.0	(1.4–2.8)	1.3	(0.6–3.3)	2.1
average	20.4		2.3		9.0		
contact 1	20.1	(14–28.6)	2.7	(2.3–3.2)	14.5	(5.5–38.1)	1.3
contact 2	12.6	(6.1–25.8)	2.8	(1.9–4.1)	12.6	(2.2–71.8)	2.3
contact 3	31.7	(20–50.2)	2.7	(2.2–3.5)	19.6	(4.7–82.1)	1.9
contact 4	21.2	(6.7–14.6)	2.4	(2.0–3.0)	2.0	(0.7–5.9)	1.4
contact 5	21.1	(14–36.8)	2.9	(2.2–3.8)	2.2	(0.5–10.2)	2.0
contact 6	23.3	(14–39.3)	2.3	(1.7–3.1)	2.7	(0.6–12.1)	2.0
average	21.7		2.9		9.0		

Individual estimates (mean and 95% confidence intervals) from simultaneously fitting *V*_*u*_ and *R*_*u*_ given by model Eq (3) to URT infectious virus and RNA data in donors and contacts from Sia *et al*. [25].

### Modeling the relationship between infectious virus titer and total RNA

We assume that the log10 URT infectious virus titer, *ν* = log_10_ *V*_*u*_ (measured in log10 TCID_50_ per mL) can be modeled as a density dependent function of the total viral RNA, *R*_*u*_ (measured in RNA per mL), as in Ke *et al*. [13] and Goyal *et al*. [23])

$$\begin{array}{c} \nu \left(R_{u}\right)=\frac{V_{m}R_{u}^{h}}{K_{m}^{h}+R_{u}^{h}}. \end{array}$$ (6)

**Fig 5:**
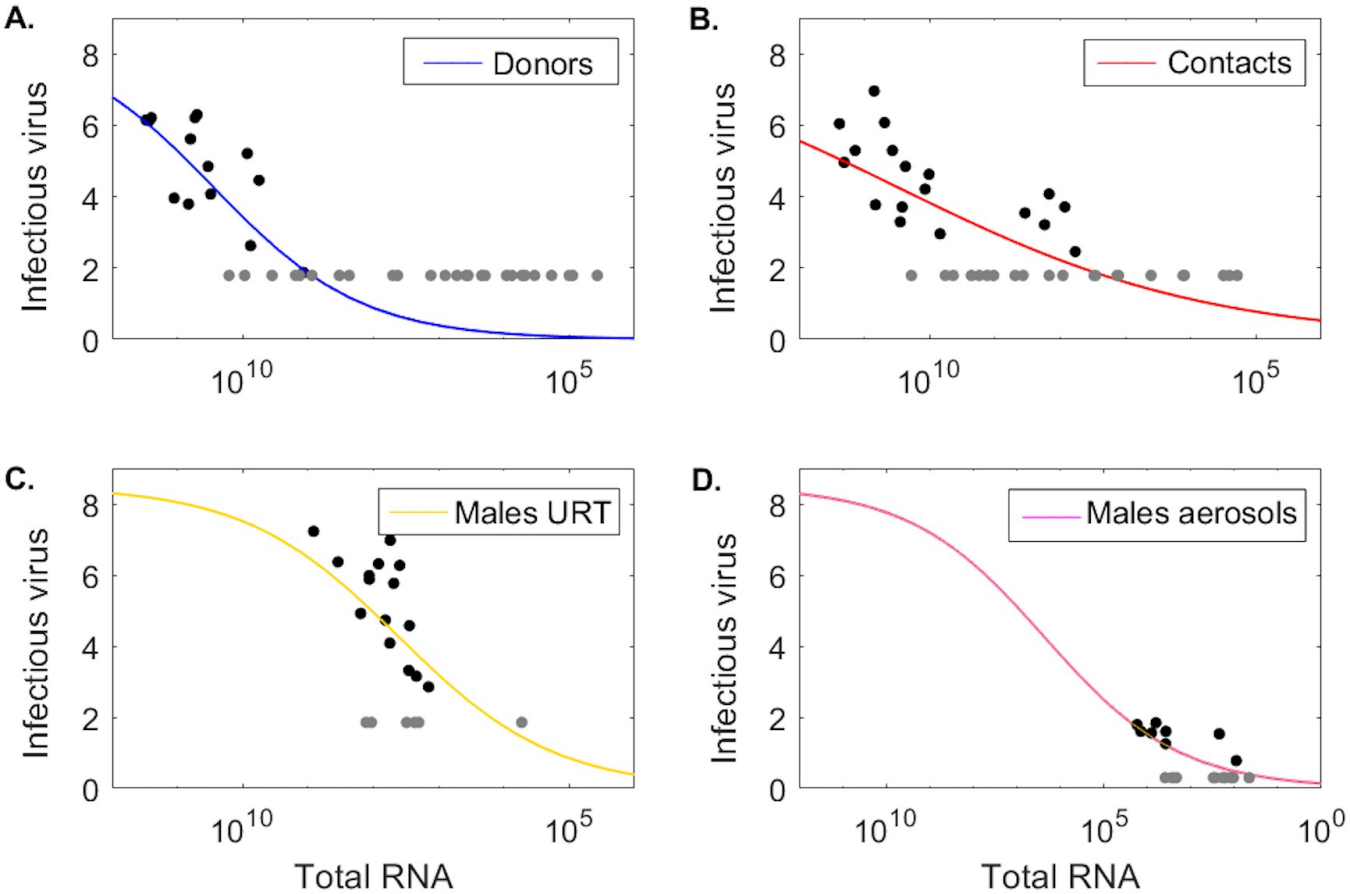
Infectious virus as a function of total RNA. Log10 infectious virus *ν*(*R*_*u*_) as a function of total RNA *R*_*u*_ given by Eq (6) versus data (circles) in (A.) donors, (B.) contacts, (C.) males URT, and (D.) males aerosols. Parameters are given in Table 3.

To avoid overfitting, we fix *V*_*m*_ = 8.5 and estimate the remaining parameters ${\text{param}}_{w}^{4}=\left\{h,K_{m}\right\}$ by fitting Eq (6) to the population (*ν*, *R*_*u*_) data from donors, contacts, males URT, and males aerosol. We excluded the female group due to limited infectious virus titers above the limit of detection. All data at and below limit of detection is treated as censored during data fitting. We maximize the functional,

$$J_{I}\left({\text{param}}_{w}^{4}\right)={\left(\sum_{j\in R_{u}^{data}}{\left(\nu \left(j\right)-\nu^{data}\left(j\right)\right)}^{2}\right)}^{1/2},$$

using the ‘nlinfit’ algorithm in matlab. The resulting population parameter means and 95% confidence intervals are given Table 3 and the population fits for log10 infectious virus, *ν* = log_10_ *V*_*u*_, versus total viral RNA, *R*_*u*_, for each group are shown in Fig 5A–5D.

**Table 3:** Population level parameter estimates.

	*h*		*K*_*M*_		ssq
Donors	0.38	(0.2–0.56)	2.8	(1.3–5.9) × 10^10^	4.9
Contacts	0.18	(0.06, 0.31)	3.0	(0.5–16) × 10^10^	6.1
Males URT	0.37	(0.01–0.7)	4.0	(1.3–12) × 10^7^	6.7
Males aerosols	0.28	(0.15–0.58)	2.3	(0.03–16) × 10^6^	1.7

Population level estimates (mean and 95% confidence intervals) obtained from fitting Eq (6) with fixed *V*_*m*_ = 8.5 to infectious versus RNA data from donors, contacts, males URT and males aerosols.

## Results

### Viral kinetics in the upper respiratory tract

**Fig 6:**
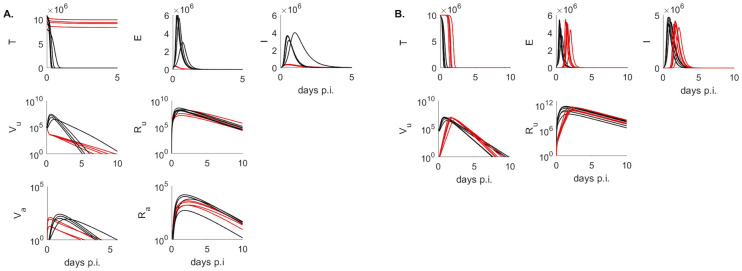
Population dynamics of model variables. Population dynamics of *T*, *E*, *I*, *V*_*u*_, *R*_*u*_, *V*_*a*_ and *R*_*a*_ as given by models Eqs 3 and 4 in (A.) males (back) and females (red) and (B.) donors (black), contacts with fixed inoculum *V*_0_ = 1 TCID_50_ (red). Model parameters are given in Tables 1 and 2.

To study the kinetics of infectious virus titers and total viral RNA in the upper respiratory tract we used a dynamical within-host target cell limitation model developed for other acute respiratory infections (Eq 3), which was normalized to include the temporal change in the subject’s weight (see Materials and methods for a full description). We estimated unknown biological parameters by fitting the model to infectious viral titer and total RNA data of four groups (males, females, donors, contacts) from two inoculation studies [24, 25] (see Materials and methods). The resulting dynamics are in good agreement with the data kinetics in all four groups, with some inter group differences in the predicted outcomes. Infectious virus titers in the male and donors groups, who were challenged with high viral dose, expand to reach a peak 12–24 hours after inoculation and decline below limit of detection by 5–10 days post inoculation (p.i.) (Fig 3A left panels and Fig 4A, red curves). By contrast, infectious virus titers in female hamsters, who were also challenged with high viral dose, are maximal at inoculation and decay below limit of detection 6–8.5 days post inoculation (Fig 3B left panels, red curves). The differences in infectious viral dynamics in the female population are due to fewer target cells getting infected compared with males and donors (*T* graphs in Fig 6A, red versus black). Total viral RNA is similar among the high inoculum groups, with peaks trending behind the infectious virus titer peaks by 6–12 hours (Figs 3 and 4 left panel, blue lines).

In the hamsters of the contact group, who were infected (rather than inoculated) the inoculum dose is unknown. We assumed that infection is started by a single virion *V*_*u*_(0) = 1 TICD50 and found that the infectious virus titers have a delayed expansion compared to the other three groups, peaking 1.5–2.4 days post infection and decaying below limit of detection 7.7–9 days post infection (Fig 4B, red curves), earlier than some of the donors. Besides the delay in viral expansion, we observe delay in infection and slightly lower overall viremia in contacts compared to donors (Fig 6B, *T*, *E* and *V*_*u*_ red versus black graphs). Interestingly, following a delay in expansion, the RNA reach similar levels in contacts and in donors (*R*_*u*_ graphs in Fig 6B red versus black curves).

We find consistent estimates between the groups for the infected cell death rate *δ*, with ranges between 1.5–5.1 per day, corresponding to infected cells life-spans of 4–16 hours. The production rate of infectious virus is highly variable among groups. In the female group we fixed the production rate to *p* = 0.1 TCID_50_/infected cell. This was significantly lower than the estimated production rates in the other groups, with contacts, donors and males average production rates of *p* = 20.4, *p* = 21.7 and *p* = 45 TCID_50_/infected cell, respectively. Moreover, the RNA production rate is variable among the two studies but similar among the groups within each study. In particular, *ρ*_1_ = 161 RNA/infected cells and *ρ*_1_ = 494 RNA/infected cells in males and females, and two orders of magnitude lower than in donors and contacts, *ρ*_1_ = 9 × 10^4^ RNA/infected cells, respectively. The differences between the studies can be noted in the higher total RNA in donors and contacts (Fig 4 blue curves), 2.5 order of magnitude higher than in males and females (Fig 3 blue curves). Overall infectious virus titers (and consequently theoretical predictions) are two fold higher in donors and contacts compared to males and females, a difference we attribute to the experimental setup in [25] versus [24].

### Viral kinetics in aerosols

In order to determine the relationship between the amount of infectious virus titer (and total viral RNA) shedding into the environment over time and host-viral dynamics in the URT, we added two viral compartments to the within-host model and normalized them to account for subject’s weight variability. The resulting within-host and aerosols model is given by systems Eqs 3 and 4 (see Materials and methods for full derivation). We estimated viral shedding parameters by validating the models against temporal aerosol data (infectious and total RNA) from the males and females (see Materials and methods). The resulting dynamics are in good agreement with the aerosol kinetics in both groups. In particular, the models predict that both infectious virus titers and total viral RNA get released into the air immediately after inoculation. For the males, the shedded infectious virus titers peak 21–37 hours after inoculation, and decay below limit of detection 3.7–5.6 days after inoculation (Fig 3A right panels, red lines). Total viral RNA peak 1.5–2.5 days post inoculation, and persist above limit of detection for the duration of the experiment (Fig 3A right panels, blue lines). For the females, the shedded infectious virus titers are maximal 3–5 hours after inoculation and decay below limit of detection 2.5–4.1 days later, faster than in the male group (Fig 3B right panels, red lines). Females total viral RNA kinetics, however, are similar to those in male, peaking 1.5–1.8 days after inoculation and persisting above limit of detection for the duration of the experiment (Fig 3B right panels, blue lines). We observe sex-specific differences in the shedding rates, with females infectious virus shedding rate *ϕ*_1_ = 6.6 × 10^−3^ being 132-times higher than that of the males, *ϕ*_1_ = 0.5 × 10^−4^ per day. The RNA shedding rates are similar among the sexes, *ϕ*_2_ = 7.3 × 10^−5^ and 11 × 10^−5^ per day for males and females, respectively.

### Basic reproductive ratio

For each group, we estimated the within-host basic reproductive number *R*_0_, which averaged at 61 for the male group, 0.3 for the female group, 44 for the donor group, and 41 for the contact group.

### Relationship between infectious virus in aerosols and transmission

Throughout the course of an infection hamsters shed both infectious and non-infectious virus into the air. Transmission to a close contact occurs when the infectious viruses reach the recipient and establish an infection. We wanted to determine whether aerosol data is a good predictor for the number of infectious virions that jump start such an infection in a close contact. While we know the exact inoculum value for the hamsters in the donor group, we do not know the inoculum value for the hamsters in the contact group. We first assumed that one infectious virus *V*_*u*_(0) = *V*_0_ = 1 TCID_50_ is sufficient to start the infection in the contact group. This lead to delayed viral expansion compared to the donor group, but similar clearance time and *R*_0_ values. Next, we investigated how these results change if we assume that a larger number of infectious virions are needed to start the infection in the contact group. We included the infectious inoculum *V*_*u*_(0) = *V*_0_ as an unknown parameter, and estimated it (together with parameters ${\text{parm}}_{w}^{2}$) by fitting model Eq 3 to the contact hamster data (Table A in S1 Text and Fig A in S1 Text). We found that the inoculum varies over a range between 40–1820 TCID_50_/ml among the six contacts. Since the contacts were co-housed with the infected donors at day 1 post inoculation, we compared the estimated *V*_*u*_(0) with the amount of infectious virus found in the aerosols of males and females. Our model predicted that the aerosol values for the infectious virus titers at day one post inoculation, *V*_*a*_(1), ranged between 96–222 TCID_50_/ml in males and between 7–52 TCID_50_/ml in females (see Fig B in S1 Text). When accounting for the fact that measured infectious virus in the Sia et al. study [25] is two-folds higher than the measured infectious virus in the Hawks et al. study [24], we find that the aerosol measurements are a good proxy for infectious inoculum. We have, however, found large confidence intervals for the estimated *V*_*u*_(0) in all contact hamsters (Table A in S1 Text), suggesting that more information is needed to determine the role of the infectious inoculum in the observed outcomes.

### Infectious virus versus total RNA levels

Detection of viral RNA by PCR testing from URT samples is the gold standard for COVID-19 diagnosis and is used to instate and discontinue control precautions, such as isolation and quarantine. However, there is no clear correlation between detection of viral RNA and detection of infectious virus titers. We use the data in the two hamster infection studies [24, 25] and our model predictions to investigate: (i) the dependence of infectious viral levels on the RNA levels, and (ii) the dynamics over time of the viral RNA to infectious virus ratio. We exclude the female group from these analyses due to limited amount of infectious virus data above the limit of detection.

To quantify the dependence of infectious viral levels on the viral RNA levels we combined all population (*R*_*u*_, *V*_*u*_) pairs. This resulted in 14 donor, 19 contact, 15 male URT, and 12 male aerosol pairs above the limit of detection and 24 donor, 17 donor 6 male URT, and 9 male aerosol pairs below the limit of detection. We assumed that the two quantities can be described by a density dependent function

$$\nu ={\text{log}}_{10}\left(V_{u}\right)=\frac{V_{m}R_{u}^{h}}{K_{m}^{h}+R_{u}^{h}}$$

(Eq 6 in **Materials and Methods**) as in [13, 23]. When we fitted this function to the population (*R*_*u*_, *V*_*u*_) data in the four groups (taking into consideration the presence of censored data, see Materials and methods for full explanation), we found that the level of infectious viruses increases sub-linearly with increases in viral RNA, with estimated exponent *h* ranging between 0.18–0.38 among the four groups. The *K*_*m*_ values are three and four orders of magnitude higher in donors and contacts compared to males URT and males aerosol, respectively, which is due to the higher RNA values in the Sia *et al*. study [25].

**Fig 7:**
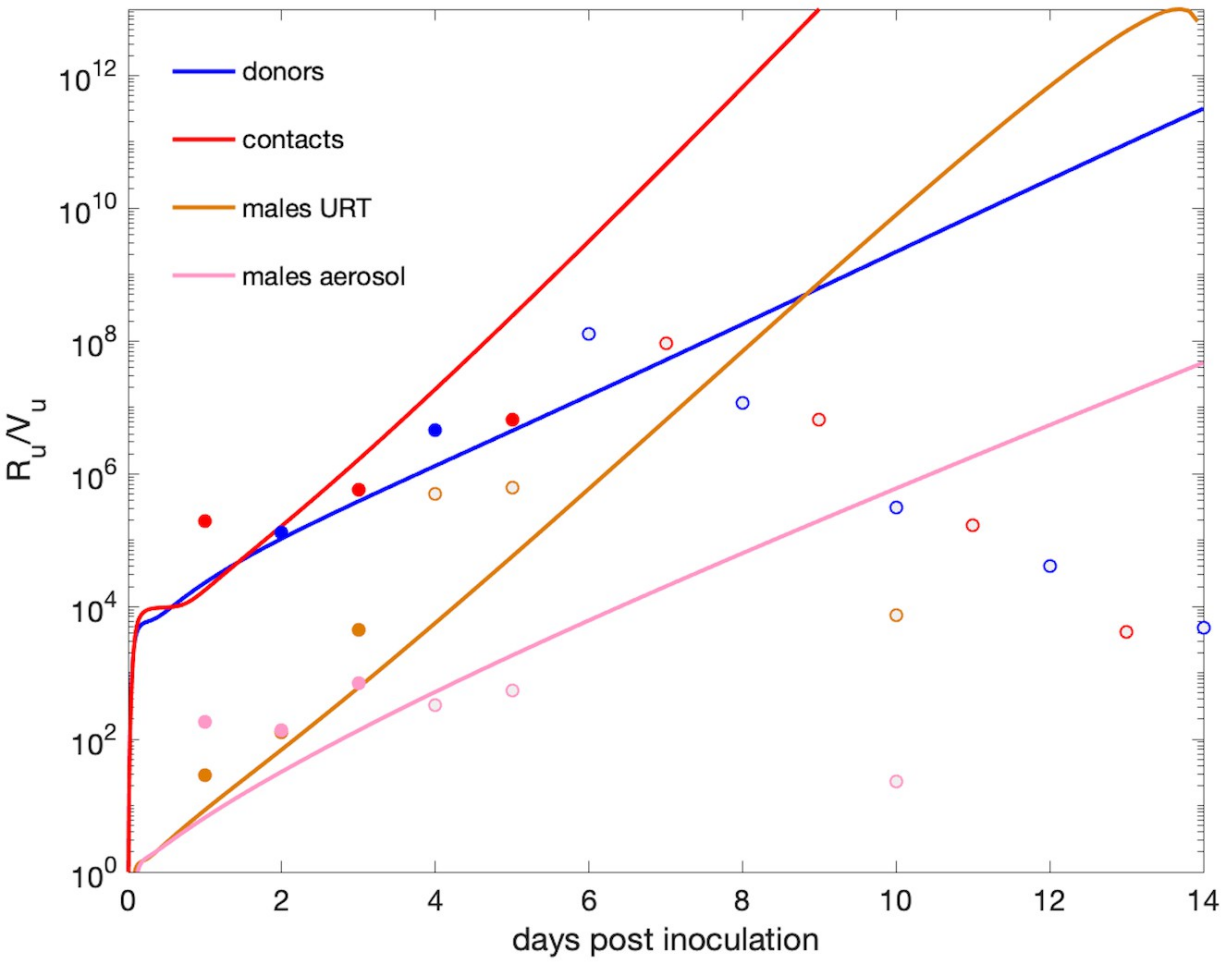
Ratio of total RNA to infectious virus over time. *R*_*u*_/*V*_*u*_ over time versus data in representative donors (blue), contacts (red), males URT (gold), and males aerosols (pink) hamsters. The light data points correspond to data where the infectious virus is at the limit of detection.

For each group, we investigated the changes in viral RNA to infectious virus ratio, *R*_*u*_/*V*_*u*_, and found it to be time-dependent, which suggests that the two measurements are explaining different biological processes. Specifically, we found that the average *R*_*u*_/*V*_*u*_ grows between 2 × 10^4^ and 10^6^ in the first 4 days in donors, between 2 × 10^4^ and 3 × 10^8^ in the first 5 days in contacts, between 8 and 600 in the first 3 days in males URT, and between 6 and 136 in the first 3 days in males aerosols, consistent with the data (Fig 7 blue, red, gold, and pink lines). Later *R*_*u*_/*V*_*u*_ ratio predictions from models Eqs (3) and (4) are no longer reliable, as the infectious virus decays below limits of detection making the ratio unrealistically high.

## Discussion

In this study, we developed within-host and aerosol mathematical models of SARS-CoV-2 infection in golden hamsters that describe the kinetics of infectious virus and total viral RNA in the upper respiratory tract and aerosols. We fitted the models to data from two studies [25] and [24] which included four separate groups: three that were challenges with high viral dose (donors, males and females) and one that was challenged through infection following close proximity with an infected host (contacts). We estimated several key parameter values for each group and determined inter group variability. We found that the within-host basic reproductive number *R*_0_ is less than one in all female hamsters, indicating limited viral spread. By contrast, *R*_0_ in above one in all male hamsters from all groups (donors, contacts, males), indicating successful viral spread. Sex-based differences have been reported in other studies, which report that favorable outcomes in females are immune mediated [28, 29]. The hamsters in the contact group had the lowest *R*_0_, averaging at 41, higher than other studies [13]. The other two groups, who were challenged with high viral dose, had slightly larger *R*_0_ estimates, with average *R*_0_ of 44 in donors and 60 in males. The death rate of productively infected cells ranged between 1.5 and 5.1 per day, corresponding to infected cells life-spans of 4 to 16 hours, similar within the groups and longer than in humans studies [13].

To model infectious virus shedding into the environment, we extended the upper respiratory tract model to account for infectious virus titer and total viral RNA emitted into aerosols. Within-host and aerosol model fitting showed that infectious virus titers get released into the air immediately after inoculation with peak shedding 21–37 hours after inoculation in males and 3–5 hours in females. Infectious viral shedding ends faster in females compared to males, with virus in aerosols losing replication-competency by 2.5–4.1 days post infection in females and 3.7–5.6 days post inoculation in males. The predicted loss of virus infectivity in aerosols before day six is consistent with experimental observations that have shown lack of transmission to naive hamsters co-housed with hamsters infected six days prior [25].

Our models and data herein predict that viral RNA persists in both upper respiratory tract and in aerosols long after replication-competent virus stops being detected, with RNA values staying above detectable levels at least a week after the infectious virus is lost. While in public health setting a SARS-CoV-2 diagnostic is determined by PCR assays, RNA levels are not always indicative of virus infectivity, with PCR specificity for detecting replication-competent virus decaying as the cycle threshold (Ct) values increase [30–32, 32]. We used the models in this study to determine the connection between viral RNA and replication-competent virus levels, and found that the ratio of viral RNA to infectious viral titers is time-dependent and ranges between 10^2^ and 10^8^ RNA/TCID_50_ in the first five days following infection, wider than in other studies [11, 12]. We also found that after day five, the RNA to infectious virus ratio is no longer a reliable measurement of infectiousness, with the measured RNA values indicating the presence of genomic fragments, immune-complexed or neutralized virus, rather than replication-competent virus [33]. These estimates may be species and disease specific, with human studies reporting variable lengths of infectious viral shedding [16, 32, 34], with larger shedding windows during severe disease [34]. However, our results are consistent with the reported lack of transmission in contact golden hamsters co-housed with an infected donor at day six [25].

We have also investigated the population level relationship between the amount of RNA and the amount of infectious virus in a sample and found that the infectious virus increases in a density-dependent manner with the viral RNA, as suggested by previous work [13, 24]. The results (consistent among the four groups considered) showed that when the viral RNA is high, the level of infectious virus saturates, consistent with our temporal results that show that it is unlikely we can predict infectiousness at high RNA:TCID_50_ ratio. The turning point where viral infectiousness starts to saturate is study dependent, with differences due to experimental settings.

Our study has several limitations. We assumed that the initial target population is *T*(0) = 10^7^ epithelial cells/ml, as in influenza [26]. This choice does not alter the results in this study, but it impacts the estimate of parameter *p*. We can obtain the same results for any other initial number of susceptible epithelial cells, as long as the *p* × *T*_0_ is fixed at the current estimates. We assumed that both infectious virus clearance and RNA degradation rates are known, with infectious virus clearance set at influenza levels *c* + *d* = 10 per day (corresponding to life-span of 2.4 hours) and the degradation rate set arbitrarily at *d* = 1 per day (corresponding to life-span of one day). Using sensitivity analysis we have found that changing the clearance rates to *c* + *d* = 15 and *c* + *d* = 5 does not influence the results (not shown). In all instances, however, the RNA degradation needs to be small, *d* = 1 or smaller, to explain the differences between infectious virus and viral RNA decay. Moreover, we had to include an additional removal of the aerosol infectious virus, which we assumed was due to infectious viral inactivation due to the elements, which we set at *d*_1_ = *d* = 1 per day. We have also considered that all neutralized virus leads to RNA production. Further information is needed to determine the biological processes leading to increased degradation of infectious virus in aerosols compared to upper respiratory tract and those leading to the persistence of RNA in upper respiratory tract and aerosols after infectious virus is lost. Lastly, due to limited aerosol data in the females (with some subjects having just one data point above the limit of detection) we could not properly identify sex-specific differences and excluded this group from some of the analyses.

In conclusion, we have developed a within-host and aerosol model for SARS-CoV-2 infection in golden hamsters and used it to investigate the dynamics of viral RNA and infectious virus titers in URT and aerosols. We validated the models against data and used it to determine the temporal relationship between infectious virus, viral RNA and the probability of a nearby host getting infected. The results can guide interventions.

## Supporting information

S1 Text Theoretical solutions and estimated parameters when *V*_*u*_(0) a variable parameter, and model predicted aerosol virus titers. (PDF)
