# Supplementary material for: Modeling within-host and aerosol dynamics of SARS-CoV-2: The relationship with infectiousness
Source: PLoS Comput Biol. 2022 Aug 1;18(8):e1009997. doi: 10.1371/journal.pcbi.1009997 (PMC9371288; doi:10.1371/journal.pcbi.1009997)
Supplement: S1 Text — (PDF) [file pcbi.1009997.s001.pdf]

Modeling within-host and aerosol dynamics of SARS-CoV-2: the relationship with infectiousness - Supplementary Material

Nora Heitzman-Breen [1] and Stanca M. Ciupe[1, *]

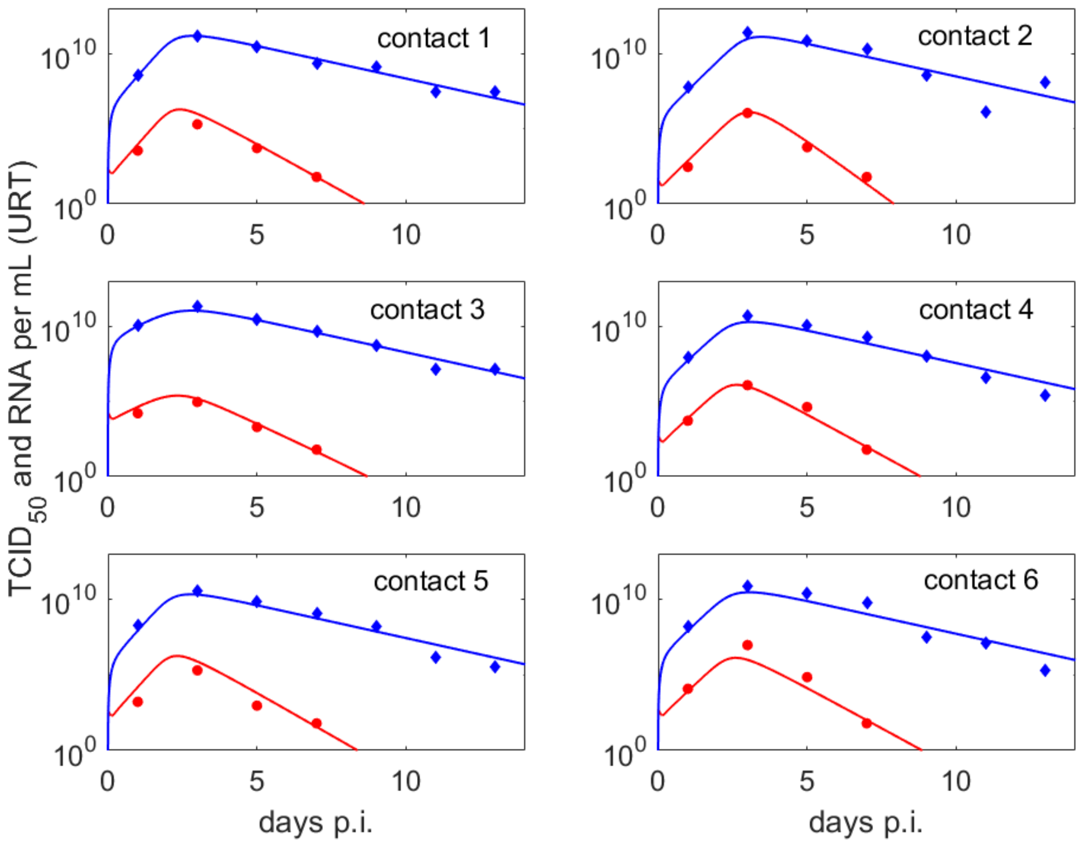

Fig A: Dynamics of infectious virus V_u (red lines) and viral RNA R_u (blue lines) as given by model Eq. (3) versus infectious viral titers (red circles) and viral RNA (blue diamonds) in the upper respiratory tract of contacts with $V_u(0) > 1$ TCID50. Model parameters are given in Table A.

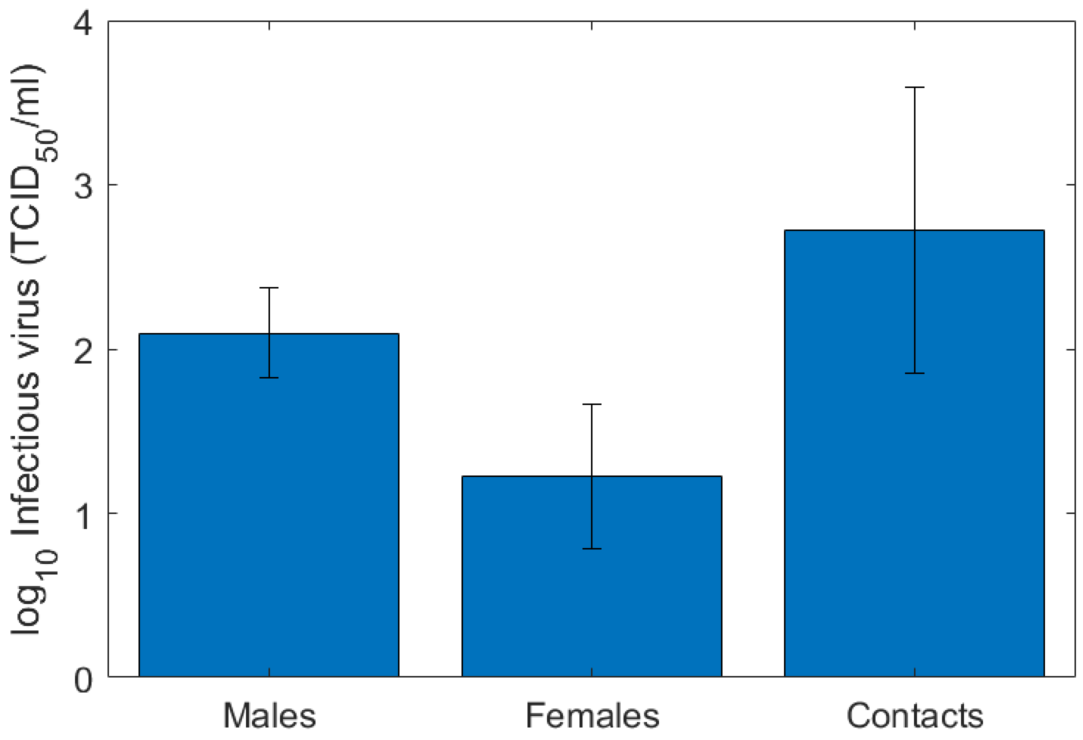

Fig B: Viral load in aerosols at day one, $V_a(1)$, given by models Eq. (3) and Eq. (4) in males and females and V_0 estimates in contacts given by Eq. (3). Model parameters are given in Tables 1 and A.

	p		δ		$\rho \times 10^4$		V_0	ssq	
c1	5.4	(0.8-38.6)	2.5	(1.6-3.9)	7.7	(2.0-30)	236	$(3 \times 10^{-1}$-$1.6 \times 10^5)$	1.1
c2	4.8	(2.1-11.3)	3.5	(1.3-5.1)	8.9	(1.4-55)	39	$(3 \times 10^{-1}$-$5.7 \times 10^3)$	2.4
c3	4.8	(4.0-5.8)	10	(8.0-12.6)	6.3	(1.6-6.2)	1820	$(4.4 \times 10^3$-$7.5 \times 10^4)$	0.9
c4	3.9	(0.5-32)	2.5	(1.4-4.5)	1.0	(0.3-4.1)	531	$(9.1 \times 10^{-1}$-$3.1 \times 10^7)$	1.3
c5	5.2	(0.2-138)	2.6	(1.3-5.1)	1.1	(0.1-9.9)	445	$(8.8 \times 10^{-3}$-$2.2 \times 10^7)$	1.9
c6	4.0	(0.5-30)	2.5	(1.5-4.1)	1.5	(0.2-10.5)	532	$(4.6 \times 10^{-1}$-$6.1 \times 10^5)$	2.1
avg	4.7		3.9		8.6		332		

Table A: Individual estimates (mean and 95% confidence intervals) from simultaneously fitting V_u and R_u given by model Eq. (3) to URT infectious virus and RNA data in contacts from Sia *et al.* when $V_u(0) > 1$ TCID50.

References

[1] Sia SF, Yan LM, Chin AW, Fung K, Choy KT, Wong AY, et al. Pathogenesis and transmission of SARS-CoV-2 in golden hamsters. Nature. 2020;583(7818):834–838.
